# The Non-Dialysis Chronic Renal Insufficiency study (ND-CRIS): an open prospective hospital-based cohort study in France

**DOI:** 10.1186/s12882-016-0307-6

**Published:** 2016-07-22

**Authors:** Jacques Massol, Gérard Janin, Camille Bachot, Christophe Gousset, Geoffroy Sainte-Claire Deville, Jean-Marc Chalopin

**Affiliations:** Institut PHISQUARE, 20, rue Saint Saëns, 75015 Paris, France; Centre Hospitalier de Mâcon, 350, boulevard Escande, 71018 Mâcon Cedex, France; Department of Nephrology, CHRU de Besançon, Hôpital Jean Minjoz, 3, boulevard Alexandre Fleming, 25030 Besançon, France

**Keywords:** Chronic renal disease, Renal insufficiency, Cohort, Epidemiology, Pharmacoepidemiology

## Abstract

**Background:**

Chronic kidney disease (CKD) amounts to a heavy burden for health services. There is no long-running epidemiological tool for CKD before dialysis. We here present the protocol for a cohort of patients with “non-dialysis” CKD receiving care in the Bourgogne-Franche-Comté region of France. The aim of this cohort was to periodically describe the characteristics of patients included and their care provision, to analyse evolution in care and patients’ kidney function outcomes.

**Methods:**

The ND-CRIS cohort is prevalent and incident. Patients are included in the cohort if over 18, with a glomerula filtration rate (GFR) <60 ml/min/1.73 m2, non-dialysed, informed on the research and not having opposed it, and followed by a nephrologist in one of the 9 centres in the region, (3 pilot centres joined by 6 others in 2015). All the patients are followed up, with varying time lapses according to the degree of GFR deterioration. Data is collected by clinical research assistants (CRAs) using a dedicated computerised case-report form (CRF). Professional practices are assessed using indicators defined by the French Health Authority. The follow-up of patients included should enable assessment of the evolution of their GFR and co-morbidities. The periodic descriptions should give insight into evolution in epidemiological terms.

**Discussion:**

The ND-CRIS meets a need in epidemiological tools in France for CKD. The cohort does claim to be representative, of ND-CKD patients receiving care from nephrologists. The open and incident nature of the cohort and the number of patients included in the ND-CRIS should provide answers to questions that cannot be answered by smaller solely prevalent cohorts. The numbers of patients included over the study period (2391 patients in 3 centres in 3 years) suggests that the figure of 5000 patients should be reached by 2017. The participation of nephrologists and the rate of inclusions point to the feasibility of the implementation of this cohort. Beyond the information to be found in the CRFs, this cohort should also enable ad hoc studies, in particular in the area of pharmaco-epidemiology, and it could later serve as a research platform and as a public health surveillance tool.

## Background

Chronic kidney disease (CKD) or renal insufficiency (CRI) is a growing burden for health services [1]. It affects 10 % of the adult population in Western countries such as the USA [2]. In France, the data is sparse and fragmented, derived from studies on high-risk populations and one prospective study running from 2004 to 2006 in the Nancy urban area (EPIRAN) which enabled an estimation of the annual incidence of CKD in the general population as being 1/1000 (1.3/1000 among men and 0.7/1000 among women) [3]. According to the French health authority (HAS) the prevalence of CKD in France is around 3 million adults, or about 10 % of the adult French population, with around 70 000 cases of ESRD (end-stage renal disease) [4].

A large body of recent data has altered the way CKD is viewed and managed, which has led to an updating of guidelines – KDIGO 2012, NICE 2008, 2014 [5–7]. Resorting to replacement systems (dialysis or renal transplant) is a major turning-point in the illness. This is why the so-called “end-stage” forms (ESRD) during which patients need to resort to renal replacement systems, are distinguished from less advanced forms, which precede these final resorts, and which are referred to as “non-dialysis” chronic kidney disease (ND-CKD). Numerous professionals are involved in the care and management of CKD, and in the early stages specialised nephrologists are not always involved. Further to this, the indicators required to refer a ND-CKD patient to a nephrologist (albuminuria and/or fall in GFR) are still in debate.

In France as in other countries, for a long time the so-called “end-stage” phase of the illness focused the attention of the health authorities on account of its weight in terms of morbidity, mortality and quality-of-life, and also on account of the cost of care, linked in particular to dialysis. Today, however, considerable interest has been aroused in ND-CKD on account of its increasing weight in public health, linked to the ageing of the general population and the increase in frequency of its main determinants, in particular diabetes and hypertension. This focus is all the more relevant because optimal care provision in the early stages of kidney disease can contribute to delaying the deterioration in kidney function, and can even avoid the need to resort to renal replacement therapies.

In 2004, the *Agence Nationale d’Accréditation et d’Evaluation des Soins* (ANAES) (then the French national agency for accreditation and evaluation of care) issued recommendations for slowing the progression of chronic kidney disease in adults [8]. At present, legislation by the French parliament is in debate concerning the implementation of pilot projects for coordinating the health itineraries of patients in the area of chronic diseases such as CKD [9].

Although since 2002 regular epidemiological data has been available on ESRD, with the REIN registry [10] and soon data from the large national prevalent cohort for ND-CKD (the CDK-REIN cohort) [11] and the NEPHROTEST cohort [12], there is in France no long-running epidemiological data system for ND-CKD, and the epidemiology of CKD is as yet not well mapped out.

With an already formed network of hospital nephrologists^a^ managing ESRD patients undergoing dialysis as well as patients before dialysis in the Bourgogne-Franche-Comté region of France, and with a funding from the *Foundation Transplantation,* we have been able to form a cohort of patients with chronic kidney disease before dialysis (the Non-Dialysis Chronic Renal Insufficiency cohort, ND-CRIS), for which the protocol is presented here. The creation of this ND-CRIS cohort originates from the demand of nephrologists in this network, who wanted to have regular data available on the populations they have to manage, on their own practices, and on the evolution of the populations that they follow in consultation.

## Objectives

The ND-CRIS cohort was designed to meet three main objectives:

First, to describe the evolution of the incidence/patient characteristics of ND- CKD patients managed by nephrologists along the time in order to gain knowledge on the epidemiological evolution of CKD in France.

Secondly, to describe the quality of the management of the CKD patients included in the cohort, related to the ANAES/HAS indicators. Alongside, the ND-CRIS cohort should also provide regular data on therapeutic management (in particular prescription of medication), and on the biological examinations prescribed by the practitioners in the centres.

Finally, to map the slopes for deterioration in renal function over time, to describe the evolution of patients included up to the time of any recourse to renal replacement therapies, with a specific description of patients reaching the “end-stage”, and to determine the risk factors related to patterns of evolution.

To enable analysis of the quality of care management, among the 10 indicators recommended by ANAES [8] we chose five that that are readily accessible since they are collected in the CRFs, and another that is recommended in the care itinerary guide issued by the HAS (*Haute Autorité de la Santé*) [4]. These indicators will enable the following:

- follow-up of the evolution of blood pressure and proteinuria means among these patients [8]
- assessment of medication-related iatrogenic risk via systematic collection and measurement of the proportions of patients receiving aminoglycosides, NSAIDs, and iodine contrasting agents [8]
- assessment of the proportion of patients receiving renin-angiotensin system antagonists, angiotensin converting enzyme (ACE) inhibitors, and angiotensin II receptor antagonists (ARA2) [8]
- assessment of the proportion of diabetic patients under ARA2 [8]
- assessment of the proportion of patients vaccinated against hepatitis B from stage 3b in CKD [4]
- quantification of the frequency of follow-up consultations for patients [8].

Beyond the information available in the CRFs, the ND-CRIS cohort will also enable ad hoc studies to assess the risk-benefit ratio of health products and procedures.

*In fine*, the ND-CRIS cohort should provide a long-running epidemiological tool in the area of ND-CKD.

## Methods/Design

The ND-CRIS is an open, prospective, prevalent, incident cohort of non-determined duration.

It is implemented in nine nephrology centres in hospitals in the Bourgogne-Franche-Comté region of France, and implicates 44 investigating physicians, assisted by five CRA, under the coordination of a project head. The project as a whole is managed by a steering committee and an head operational director.

**Fig. 1:**
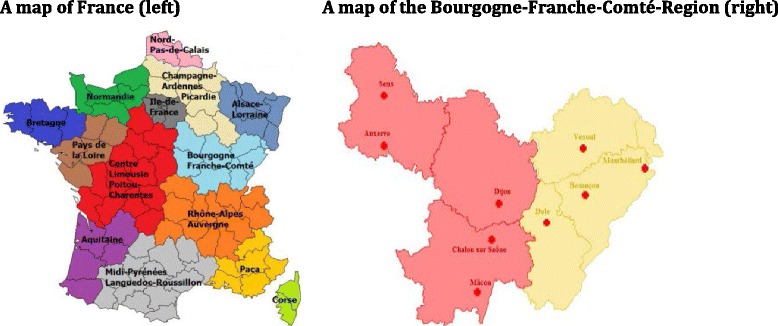
Geographical situation of the Bourgogne-Franche-Comté investigating centres

The investigating hospitals are located in Auxerre, Besançon, Chalon sur Saône, Dijon, Dôle, Mâcon, Montbéliard, Sens and Vesoul (see Fig. 1). A feasibility or pilot phase was conducted in three centres (Besançon, Mâcon and Belfort-Montbéliard) between April 2012 and June 2015. The feasibility of the data collection and the organisation of the work in these facilities was thus explored and improved. In this period, all eligible patients already followed up in the centres by nephrologists were included (prevalent patients) as well as all new patients meeting the inclusion criteria as they were taken on (incident patients). So far, the extension phase has enabled eight of the nine centres in the region to get underway, the 9^th^ being set for December 2015.

### Selection and inclusion of patients

Eligible subjects are those presenting CKD with two GRF measures <60 ml/min/1.73 m^2^ calculated by the MDRD method. This population is stratified according to the operational definitions of CKD provided by the KDOQI guidelines [13] (stages 3 to 5: CKD stage defined as estimated glomerular filtration rate ≥45 (stage 3a), 30–44 (3b), 15–29 (4), and <15 (5) mL/min/1.73 m^2^) and non-dialysed. The population is composed of adults (>18 years) managed by nephrology teams in the participating centres.

Following information delivered to all eligible subjects, patients were included in the cohort if the second GFR measure was still <60 ml/min/1.73 m^2^, provided they did not refuse to participate. Patients who had received a transplant or were under dialysis, patients temporarily in one of the centres but not followed up there, and patients unable to understand the information sheet were excluded.

Patient follow-up: all patients whose GFR was <60 ml/min are systematically followed in consultation, with a frequency mainly dependent upon the degree of deterioration of the GFR and the co-occurrence of proteinuria. Certain patients with a GFR between 45 and 60 ml/min and whose condition does not warrant specialist nephology care are in general referred to their treating physician, with instructions to refer them back should their condition deteriorate, or in case of specific renal problem.

### Data collection and management

The data collection is performed by the 5 CRAs in the participating centres from medical files. The actual organisation of the data collection in each centre depends on the internal functioning of each facility. The hospital manager, the medical information department (DIM) and the head physician in each department issue the CRAs with the authorisations required for access to the medical files in paper and/or electronic form.

The CRAs have the task of identifying the patients eligible for the cohort on the basis of the coming consultations by physicians in the centres. The physicians present the cohort to their patients and refer back to the CRAs to inform them of any refusals to participate in the cohort. Information on refusals can be indicated on the consultation report, on the patient’s electronic file, or by other means chosen by the physician in charge. Once the patient is informed, and provided he/she has not expressed refusal, the CRA initiates data collection from the medical file. At each new consultation, the CRA collects the new data on the consultation and on hospitalisation provided in the medical file. For patients with GFR <60 ml/min at inclusion who have not consulted again 18 months later, the CRA investigates the reasons from the healthcare teams and, if required, from the person’s birthplace (to ascertain vital status). Patients are considered lost to follow-up if this procedure does not enable any information to be obtained concerning the patient.

The CRAs transfer all relevant information on the cohort to the medical teams.

The data is collected using an electronic CRF developed by SLC Expertise. The CRF is created under Microsoft Access, a relational database management system. Collection, processing and storage of data complies with ethical and good epidemiological practice recommendations, ADELF, ADEREST, AEEMA, EPITER (France 2007) [14] and with the ISPE Guidelines for Good Pharmaco-epidemiology Practices (GPP) [15] (Table 1).

**Table 1:** List of collected data

Data collected	At screening	At inclusion	At follow-up consultations	At removal from the cohort
Age /Gender/ « department » socioprofessional status	X			
Data of consultation	X	x	X	
Creatinine levels	x	x	X	
Diagnosis of kidney disease		X		
Risk factors, complications, hospitalisations		x	X	
Clinical examination : weight, stature, blood pressure		x	X	
Biological investigations : proteinuria, microalbuminuria, calcemia, phosphoremia, hémoglobin, 25 OHD3, PTH, ferritinemia, iron, saturation, CRF		x	x	
Investigations, Imagery with contrasting substances			x	
Medication		x	x	
Date of dialysis, method of dialysis, date of fistula instatement, patient information				x
Date of transplant, patient information				x
Death and cause				x
Other reason for removal from the cohort				x

### Inclusion potential and activity of centres

One of the objectives of the ND-CRIS cohort is also to provide the hospitals with information on their patient number. In January 2015, the estimations of annual number of patients for this pathology conducted in collaboration with heads of nephrology departments were as follows (Table 2).

**Table 2:** Estimation of CKD patients by centre

Auxerre:	500 patients	Besançon:	1,000 patients
Chalon sur Saône:	500 patients	Dijon:	1,000 patients
Dôle:	400 patients	Mâcon:	800 patients
Montbéliard:	900 patients	Sens:	250 patients
Vesoul:	600 patients		

### Statistical analyses

Patient numbers and characteristics will be described for the overall cohort, and for each centre. Data analysis will concern the variables collected, and descriptive statistical analyses will be performed each year on all variables.

With 5,000 patients, assuming equal groups, an average follow-up of 5 years, and a censoring rate of 0.1 per year, the minimum detectable hazard ratios (with two-sided α = 5 % and 80 % power) for a survival analysis are 1.20, 1.15 and 1.12 for baseline event rates of 0.05, 0.10 and 0.20 per year [12].

Depending on their nature, quantitative variables will be described by total numbers, numbers of missing data, numbers of items of data collected, median, mean and standard deviation, 95 % confidence interval and extreme values (max/min).

Qualitative variables will be described in terms of total numbers, numbers of missing data, numbers of items of data collected, percentages for each modality of response (calculated from completed data alone) and 95 % confidence interval.

The statistical tests will be unilateral and the significance threshold is set at 5 %. Inter-group comparisons will be performed using:

- the Chi^2^ test or the Fischer exact test if the theoretical numbers are below 5 for qualitative variables,
- Student’s test for Gaussian quantitative variables
- Wilcoxon’s non-parametric test for semi-quantitative or non-Gaussian quantitative variables.

The statistical analyses will be performed on SAS® software, version 9.4, SAS Institute, NC, Cary, USA.

The evolution of the main variables will be calculated on the basis of data from as close as possible to January 1^st^ of year N and year N + 1. In each centre and for the overall population, patient incidence and prevalence will be measured. For each category of treatment, the percentages of patients currently exposed and previously exposed will be calculated. The number of pharmacological treatments prescribed simultaneously will also be calculated.

The number of consultations will be recorded for each centre and for the different stages of kidney disease. Biological parameters, GFR, BMI and blood pressure will be recorded for the overall population, for the population in each centre, and for the different stages in kidney disease (3, 4, or 5).

Likewise, over one year, the percentages of patients whose GFR improves, stabilises or deteriorates are also calculated.

The main indicators derived from the cohort are issued three-monthly to the medical teams. A complete report presenting all the variables described in the CRFs will also be sent to the centres once a year. There will be regular publications of results in scientific journals.

Quality control procedures will be implemented yearly in each participating centre. This will include a complete audit of ten randomly-selected medical files.

The data collected via this cohort is indirectly identifiable, thus requiring the authorisation of the French advisory committee on information processing in the area of health (CCTIRS), equivalent to an ethics committee [16], and of the CNIL (French data protection authority). The CCTIRS issued approval on July 27^th^ 2015 and the CNIL on November 26^th^ 2015.

For this cohort, an information sheet is issued to the patient by the physician.

## Discussion

The need to develop epidemiological cohorts in France has been underlined by the Government and by health product manufacturers [17]. Their value and function, and the quality criteria to be met for them to yield tools for public health monitoring, were described in a document issued by InVS in 2010 [18].

The ND-CRIS is a large cohort that complements the existing epidemiological tools available in France in the area of kidney disease, in particular the REIN registry [10] and the CKD-REIN Cohort [12]. It takes its place among the large international cohorts on CKD, such as those reviewed in the Chronic Kidney Disease Prognosis Consortium [19].

While the REIN registry collects national data in France concerning patients with end-stage renal disease (ESRD), the ND-CRIS concerns the same population as the CKD-REIN cohort on regional scale. The size, the objectives and the methods of these two cohorts differ. While the CKD-REIN is a closed cohort of prevalent patients (around 3600 expected) aiming for national representativeness, the ND-CRIS cohort is open and concerns both prevalent and incident patients.

In the CKD-REIN the patients are to be followed over 5 years, while for the ND-CRIS patient follow-up will be pursued until death or until the instatement of renal replacement therapy.

Finally, while the main aim of the CKD-REIN is to identify risk factors and markers for the progression of chronic kidney disease from a defined sample, the aim of the ND-CRIS is to provide an on-going epidemiological tool to gain knowledge on epidemiological trends, and evolution in the quality of care management for patients with ND-CKD, as well as to enable ad hoc studies on various subjects of interest. Because it seeks to address research questions that differ from those of the CKD-REIN cohort, the ND-CRIS cohort widens the field of research in the area of chronic kidney disease.

The ND-CRIS cohort has several strengths. The type of cohort – open, incident/prevalent and without a time limit – means it is expected to provide answers to questions that closed cohorts cannot address, in particular because of their lack of temporal representativeness. The participation of nephrologists across the Bourgogne-Franche-Comté region ensures territorial representativeness for CKD patients cared for by a nephrologist. It could thus provide a tool for regional public health policies. It will also have temporal relevance.

The results of a pilot study conducted in three of the nine nephrology centres, which are to be published, have demonstrated the feasibility of the ND-CRIS cohort, meeting adequate quality requirements. At present 8 of the nine centres have started up, and all will be underway in February 2016. 2391 patients have been included and their follow-up has been initiated. The number of patients that can be reasonably expected given the turnover of patients followed by the nephrologists concerned (at least 5000 patients by 2017), in addition to providing routine descriptive data from the CRFs, will also enable ad hoc studies.

The ND-CRIS cohort also has certain limitations. As indicated earlier, the ND-CRIS does not a priori claim national representativeness. The management of patients with kidney disease varies from country to country and region to region. Thus the regional character of the ND-CRIS will preclude any extrapolation of the data, which will be considered as dependent on the region or on the care management implemented. The confrontation of the characteristics of the ND-CRIS patients with those in the CKD-REIN cohort could however provide information on the degree of national representativeness of the ND-CRIS cohort.

The ND-CRIS is not representative either of all ND-CKD patients. Indeed, screening and follow-up of patients in the early stages of the disease are generally managed by GPs or other specialists (diabetologist, cardiologist …). The ND-CRIS does however aim to be representative of ND-CKD patients managed by nephrologists, who according to HAS recommendations [4] should correspond to a ND-CKD population with a creatinine clearance of <45 ml/min, and a population with a clearance between 45 and 60 ml/min in case of rapidly progressing or complicated forms, or in case of doubt as to the nature of the renal disease.

Although the CKD-EPI calculator has shown better performances in classifying and predicting evolution in kidney disease, in particular for GFRs between 45 and 59 ml/mn [19], and for threshold values around 60 ml/mn, the MDRD has been chosen because it is the most widely used by nephrologists in the region, and is the equation at present recommended by HAS. In addition, as classification errors generally lead to a reclassification of lesser severity of impairment of renal function, the MDRD does not lose CKD screening ability. The conversion of MDRD values to CKD-EPI values will however be performed *a posteriori*. Cystatin C titration, however, since it is not covered by health insurance reimbursements, cannot be used.

Like all cohorts with long-term follow-up, one of the most problematic limitations is the number of subjects lost to follow-up. Although unavoidable, loss to follow-up cannot be precisely estimated over the years, but it could be reduced by the CRAs referring back to the nephrologists, and if need be to the place of birth of missing subjects to ascertain vital status.

Finally, at the present stage, the ND-CRIS does not yet have a biobank as does the CKD-REIN. The nature of the ND-CRIS cohort and its scale mean that it would be difficult to collect and store samples. In addition, the immediate objectives of the ND-CRIS differ from those of the CKD-REIN, and do not in fact require a system of this sort. It would nevertheless be possible to envisage the creation of a biobank in an ancillary protocol.

For the moment, the ND-CRIS cohort will provide nephrologists with regular data on the epidemiology of the population they are managing, and on their own practices. Each participating centre will receive the data on its own practices, but only aggregated data will be used in communications and publications. With the follow-up of included patients, the routine data contained in the CRFs will enable the trends in the characteristics of the population managed for ND-CRIS to be documented, and also, allowing for an adequate time-lapse, the impact of management on renal function, on complications and on recourse to renal replacement therapies.

The ND-CRIS can already afford scope for ad hoc studies on subjects of interest, such as pharmaceutical products or other health products.

Pharmaco-epidemiological studies are among the issues that require urgent attention on account of their scale. Indeed, the CKD population is vulnerable – a vulnerability correlated overall with the degree of deterioration in renal function – and exposed to numerous medications, which increases iatrogenic medication-related risk. In addition, the risk-benefit ratio of health products used among these patients is not well known, in particular in the moderate to severe stages of kidney disease, as they are often excluded, or not well represented, in trials. Thus the product summaries of numerous medications include a mention of particular caution required without any real evidence to back it up, so that the prescription of such substances is fraught with uncertainty. With the exception of medications with restricted indications, the large size of the cohort and the degree of exposure to a wide range of medications should in many cases enable experimental approaches to provide answers to questions on medication administered to subjects with kidney disease.

The ND-CRIS cohort should lead on to further studies and developments. We are already planned a study of patient care itineraries so as to identify barriers to optimal care in reference to health authority guidelines [4].

Finally, the ND-CRIS cohort, as an on-going epidemiological tool, could broaden its objectives to the surveillance of health safety issues.

## Abbreviations

ACE: Angiotensin converting enzym inhibitor
ADELF: Association des Epidémiologistes de Langue Française
ADEREST: Association pour le Développement des Études et Recherches Épidémiologiques en Santé Travail
AEEMA: Association pour l’Etude de l’Epidémiologie des Maladies Animales
ANAES: Agence Nationale pour Accréditation et l’Evaluation des Soins
ARA2: Angiotensin 2 Renin Antagonist
CCTIRS: Advisory Committee for Data Processing in Health Research at the French Research Ministry (equivalent to an Ethics Committee)
CH: Hospital Centre
CHRU: Regional University Hospital Center
CKD: Chronic kidney disease
CNIL: Comité National de l’Informatique et des Libertés (French data protection authority)
CRA: Clinical Research Associate
CRF: Case report form
CRI: Chronic renal insufficiency
DIM: Medical Information Department
EPIRAN: Epidémiologie de l’Insuffisance Rénale dans l’Agglomération Nancéienne
EPITER: Association pour le développement de l’épidémiologie de terrain
ESRD: End stage renal disease
GRF: Glomerular filtration rate
HAS: Haute Autorité de Santé (French National Authority for Health)
InVS: Institut National de Veille Sanitaire (French Institute for Public Health Surveillance)
ISPE: International Society for Pharmacoepidemiology
MDRD: Modification of diet in renal disease
ND-CRIS: Non-dialysis chronic renal insufficiency study
PHISQUARE: Public Health Impact Institute
